# Efficacy of a web application for stress management among Iranian college students during COVID-19 outbreak: a study protocol for randomized controlled trials

**DOI:** 10.1186/s13063-020-04949-0

**Published:** 2020-12-14

**Authors:** Fatemeh Khademian, Azam Aslani, Ramin Ravangard, Peivand Bastani, Mohammad Nami, Peyman Jafari

**Affiliations:** 1 Student Research Committee, Department of Health Information Management, School of Management and Medical Information Sciences, Shiraz University of Medical Sciences, Shiraz, Iran; 2 National Agency of Strategic Research in Medical Education, Tehran, Iran; 3 Health Human Resources Research Center, School of Management & Information Sciences, Shiraz University of Medical Sciences, Shiraz, Iran; 4 Health Human Resources Research Center, School of Management and Medical Information Sciences, Shiraz University of Medical Sciences, Almas Building, Alley 29, Qasrodasht Ave., Shiraz, Iran; 5 Department of Neuroscience, School of Advanced Medical Sciences and Technologies, Shiraz University of Medical Sciences, Shiraz, Iran; 6 Neuroscience Center, Instituto de Investigaciones Científicas y Servicios de Alta Tecnología (INDICASAT AIP), City of Knowledge 084301103, Panama City, Panama; 7 Department of Cognitive Neuroscience, Institute for Cognitive Science Studies, Pardis, Tehran Iran; 8 Department of Biostatistics, Faculty of Medicine, Shiraz University of Medical Sciences, Shiraz, Iran

**Keywords:** Randomized controlled trial, Telemedicine, Stress, COVID-19, Mobile health, College student

## Abstract

**Background:**

The prevalence of mental health disorders is increasing globally, and the prevalence of COVID-19 has made it worse. Evidence has indicated a major mental health burden and elevated anxiety associated with the new coronavirus outbreak in the general population. This study aims to evaluate an evidence-based web application (Naranj) for stress management among Iranian college students.

**Methods and design:**

This study aims to present a protocol related to a randomized controlled trial among Iranian college students. The study will be conducted on 100 students from two colleges of Shiraz University of Medical Sciences in Iran. The participants will be randomly assigned to the intervention and control groups. The intervention group participants will be provided with a web application, whereas the control group ones will be provided with an app unrelated to stress management. The primary outcome for this study will be the Perceived Stress Scale, and the two groups will be compared with respect to stress level and sleep quality.

**Discussion:**

A web application will be developed according to psychological theories and will be scientifically approved for managing college students’ stress and improving their sleep quality during the COVID-19 outbreak.

**Trial registration:**

Iranian Registry of Clinical Trials IRCT20160427027647N2. Registered on 14 May 2020

## Background

A new infectious respiratory disease, named COVID-19, appeared in Wuhan, Hubei province, China, and it has affected almost all other countries throughout the world. According to the World Health Organization (WHO, 6 July 2020), COVID-19 has affected over 11,327,790 people and has killed 532,340 people in more than 200 countries worldwide [1]. A study found that similar to the SARS-induced psychological burden [2], anxiety symptom levels increased when COVID-19 occurred in China [3]. Moreover, initial studies indicated a major mental health burden among the public, increased depressive symptoms, decreased sleep quality, and elevated anxiety during the COVID-19 outbreak [3–5]. In this regard, a study in China revealed that stress, anxiety, and depression were more prevalent among students and healthcare professionals in comparison with other professions [6]. Moreover, another study showed that Chinese young people and those who spent more time thinking about COVID-19 were more at risk [3]. The probable cause of mental health problems during the coronavirus pandemic could be related to the “hypochondria concerns” theory that means being worried about being infected [7]. According to a Spanish study, it could also be related to important life changes in a very short period of time, including changes in financial or occupational status, restrictions on movement, and cancelation of important activities [8].

In general, the prevalence of mental disorders is increasing globally [9]. Statistics have shown that about 970 million people worldwide suffered from some sort of mental illnesses in 2017 [10], 75% of whom lived in low- and middle-income countries [11]. Mental health problems are one of the main causes of the global disease burden, such a way that they account for 13% of the total global burden of disease [12]. Mental illnesses also account for 25.3% and 33.5% of all years lived with a disability in low- and middle-income countries, respectively [12]. According to the latest Ministry of Health nationwide study regarding mental health disorders in Iran in 2015, about 23.4% of the population were suspected to suffer from a kind of mental disorder. According to this report, about one out of every four people was suspected to suffer from mental disorders [13]. These results were in line with the global average [14].

It has been claimed that the prevalence of anxiety and depression has increased by about 40% over the past 30 years [15]. It has also been estimated that around 4% of the population had an anxiety disorder [16]. In addition, stress has been classified as the health epidemic of the twenty-first century by the WHO [17]. Stress has been defined as the body’s response to painful situations that cause discomfort, which can lead to depression and health problems [18]. In Iran, limited research has been conducted in this field among the general population. The results of a study conducted among the people aged 18 years in Tehran (2017) showed that more than 80% of the people had experienced at least one severe episode of stress during the past year [19]. The results of another study conducted on 10,000 adult residents of Yazd Province revealed that 34.8% of the people had experienced stress [20]. Furthermore, previous research showed that college students had experienced high levels of stress. Studies have also reported some levels of stress and anxiety among students in China (74.3%), Saudi Arabia (63.5%), Bangladesh (61%), and Egypt (59.9%) [21–24]. A case report performed in Iran also indicated that 83% of the students had experienced stress [25]. The most common stressors among the students were parental expectations, university/life balance, financial/work balance, living away from home, sleeping difficulties, and academic issues [26, 27].

Stress can lead to serious health problems and can damage almost all body systems [28]. Studies have found that stress was associated with an increased risk of rheumatoid arthritis [29], cardiovascular disease [30], cancer [31], and autoimmune diseases such as systemic lupus erythematosus, type 1 diabetes, and multiple sclerosis [32].

Despite the importance of mental health problems, WHO reported that nearly two thirds of people with a known mental health problem never sought professional help. It was also stated that stigma, discrimination, and ignorance prevented people from receiving treatment and care [10]. Therefore, societies should provide people with an easy, inexpensive, affordable, and reliable way to access medical services remotely. Digital technology initiatives, such as m-Health, could be helpful in this regard. M-Health refers to the utilization of mobile phones and other wireless technologies in medical care. Some studies have shown evidence supporting the effect of m-Health on different fields of health [33–35]. The good news is that from more than 15,000 mobile apps, at least 29% have been designed in the field of mental health [36]. The bad news is that a small number of apps are evidence-based [37]. Besides, a study that appraised mental health apps showed that only few of them could be personalized [38]. Moreover, the results of a study demonstrated that more than 50% of students reported low levels of eHealth literacy [39]. Therefore, users have to be empowered to use digital technologies effectively.

The results of a survey revealed that college students had higher anxiety levels compared to the general population after the outbreak of COVID-19. This implied that COVID-19 had negative psychological impacts on Chinese college students’ anxiety levels [40]. Considering the spread of mental health problems and the prevalence of COVID-19 that has made it worse, it is necessary to take appropriate and timely measures. In this context, mental health support should be provided and continued for all people, especially the vulnerable groups. Such support should include accurate information as well as training of suitable coping strategies to manage the situation. Therefore, the present study aims to evaluate an evidence-based web application (Naranj) for stress management among Iranian college students during the COVID-19 outbreak. It has been hypothesized that the application is effective in decreasing college students’ psychological stress. The secondary expected outcome is the improvement of sleep quality.

## Methods/design

### Aims and objectives

This study aims to assess the efficacy of a web application (Naranj) for stress management among Iranian college students during the COVID-19 outbreak.

### Design and setting

This is a non-blind, parallel-group (ratio 1:1), randomized controlled trial (superiority framework). The participants will be recruited from two colleges of Shiraz University of Medical Sciences in Iran: (1) School of Management and Medical Information (Health Information Management, Medical Informatics, and Health Services Management) and (2) School of Health.

### Sample size

The sample size is calculated based on a similar previous study [41] where the mean (SD) of perceived stress in groups 1 and 2 were 7.43 (2.93) and 9.49 (3.06), respectively. This study was a research aimed to evaluate the efficacy of a stress management app for college students. We chose this randomized controlled trial study because it was very similar to our study in terms of the intervention, outcome, and participants. After that, the sample size is calculated using the NCSS software. The minimum sample size based on power = 80%, and *α* = 0.05 is estimated at 35 individuals in each group. In order to compensate for the loss to follow-up, we will increase the sample size to 50 individuals in each group.

### Participants

#### Inclusion criteria

Students will have to meet the following eligibility criteria to be included in the study: (1) aging 20–35 years; (2) being a BSc (second and higher semesters) or an MSc student; (3) having a smartphone, computer, laptop, or tablet; (4) not suffering from known psychological problems; (5) not consuming psychiatric drugs; (6) not being addicted according to the students’ self-expression; (7) not having chronic physical diseases (including diabetes, hypertension, hypothyroidism, hyperthyroidism, asthma); (8) not having physical disabilities (including blindness and deafness); (9) being willing to participate in the study; and (10) signed informed consent forms.

**Fig. 1:**
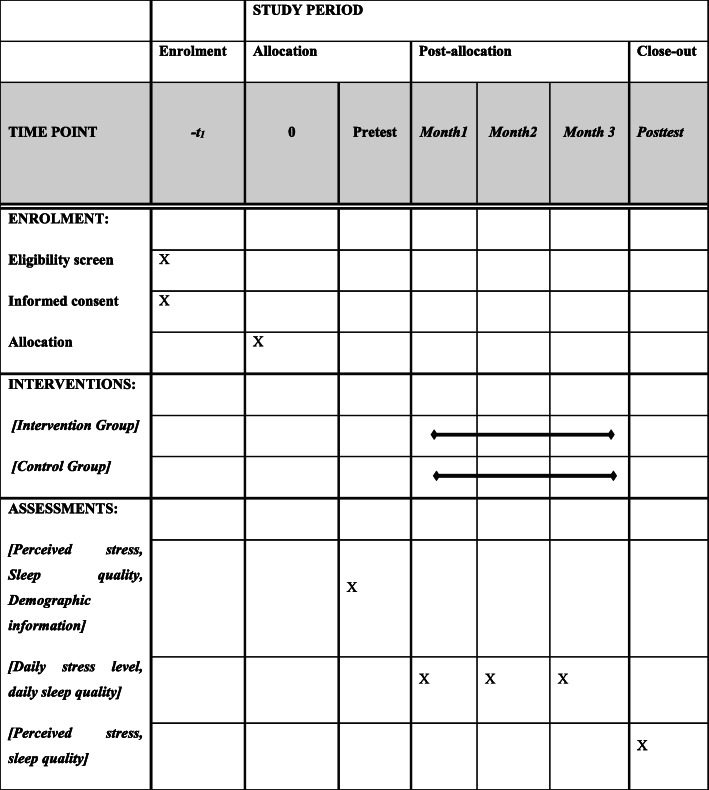
The participants’ timeline

#### Exclusion criteria

The students will be excluded if they are not willing to take part in the research.

### Randomization

The participants will be selected by the systematic random sampling method based on two separate lists of all eligible BSc and MSc students in both colleges. We will select 50 students at each college. Then, the selected students will be provided with an explanation of the aims and scope of the project and will be orally invited to participate in the research. If they accept the invitation, they will be asked to sign informed consent forms. However, if they do not give informed consent, the next student in the systematic random sampling list will be substituted. Then, we will allocate the participants of each college into 2 groups of intervention and control based on two separate 1:1 permuted block randomization lists, so that five blocks of size 10 in each college will be formed. The randomization sequence will be generated by the biostatistician using the Random Allocation Software with a 1:1 allocation. Assigning participants to their groups will be carried out via random allocation.

### Blinding

Due to the nature of the study, the existence of the two arms in the experiment cannot be concealed (this will be a study limitation), but some levels of blinding will be considered. For example, the participants on each arm will not be informed about what the other arm will receive.

### Procedures

All the participants will fill out the demographic data and other study questionnaires electronically. Then, the researcher will send a link to the intervention group to have access to the stress management app (Naranj) and another link to the control group to download an app unrelated to stress management.

The Naranj app includes a user profile, stress management strategies, consultation requests, and COVID-19 sections. The user profile includes account data, demographics, and health history (nutrition, physical activity, smoking, diseases, drug and alcohol consumption, etc.). Stress management strategies are based on the Acceptance and Commitment Therapy (ACT), Stress Reduction Theory (SRT), and some other evidence-based strategies. This section consists of various parts, including metaphors based on ACT, 4-7-8 breathing technique, Emotional Freedom Technique (EFT), pictures of nature, relaxing music, relaxing videos, educational quotes, my diary, forum, and meditation. In “my diary,” students have a space to enter their thoughts, emotions, and experiences of using the strategies. The “consultation request” section provides users with the opportunity to use emergency (receiving service during 24 h) and usual (receiving service during 48 h) consultations with psychological consultants. The “COVID-19” section provides educational content on COVID-19. In addition, this section is hyperlinked to the “stress management strategies” section. The Naranj app includes “privacy policy and terms of use,” app tutorial, contact us, and frequently asked questions, as well.

An app regarding calorie uptake management will be offered to the control group. This app is comparable to the Naranj app in terms of the need for daily use by the user. However, it is different from Naranj in terms of content. The rationale for using this app is to determine whether there will be an improvement in the outcome measures due to the stress management content in the Naranj app. Overall, both groups are provided with the same conditions. In this way, the effect of the confounding factors, if any, can be controlled and the Hawthorne effect will be minimized. According to the Hawthorne effect, individuals will alter their behaviors in response to being studied and usually refer to positive changes [42].

Both groups will have access to their specified apps for 3 months. All participants in the intervention and control groups will be requested to complete the questionnaires again after completion of the intervention.

### Data collection

The participants will be asked to fill out three electronic questionnaires. At baseline, they will be asked to complete the demographic information form, Perceived Stress Scale (PSS), and Pittsburgh Sleep Quality Index (PSQI). After the completion of the intervention, they will be requested to complete the PSS and PSQI again (Fig. 1). Since the Iranian government has advised the public to minimize face-to-face interactions and isolate themselves at home during the COVID-19 pandemic, the electronic versions of the questionnaires will be used. Information on the app use, such as daily stress level and daily quality of sleep measured daily using the visual analog scale (VAS) in the web app, as well as the frequency of using different app sections will be collected, as well. Data on the app use and the possible use of other apps, including apps related to stress management, will also be collected.

### Outcomes

#### Primary outcome measure

The primary outcome of the study will be perceived stress, which will be assessed using the Perceived Stress Scale (PSS). PSS is a valid and reliable questionnaire used to measure stress among Iranian college students [43]. PSS contains 14 items scored based on a 5-point Likert scale ranging from 0 (never) to 4 (very often). Items 4, 7, 9, 10, and 13 are scored reversely. The total score of PSS will be obtained by adding all the items. The scores can range from 0 to 56, with higher scores representing the higher perceived stress.

#### Secondary outcome measure

The secondary outcome of the study will be sleep quality, which will be evaluated using the Pittsburgh Sleep Quality Index (PSQI). PSQI is a valid and reliable questionnaire used to measure stress among Iranian college students [44]. PSQI contains 18 items on a 4-point Likert scale ranging from 0 (not at all) to 3 (three or more times a week). These items index in seven subscales: subjective sleep efficiency, sleep latency, sleep duration, sleep quality, sleep disturbance, sleep medication use, and daytime dysfunction due to sleepiness. The total score ranges from 0 to 21 where higher scores indicate worse sleep quality. A PSQI score > 5 indicates poor sleep quality.

### Statistical analysis

The Kolmogorov-Smirnov test will be used to test the normal distribution of the data. The comparability of the intervention and control groups will be assessed at baseline. The quantitative variables will be described using mean and standard deviation if they follow a normal distribution and using median and range in case of non-normal distribution. The qualitative variables will be presented as percentages and confidence intervals. In order to compare the study groups regarding the mean scores of stress and sleep quality, the independent samples *t* test will be applied. In addition, the Mann-Whitney *U* and chi-square tests will be used to compare the two groups regarding non-parametric and categorical variables, respectively. In case of confounders, analysis of covariance or linear regression model will be used. Moreover, the paired samples *t* test will be utilized in order to compare the mean scores of stress and sleep quality in each group. Intention-to-treat analysis will be employed, as well. All analyses will be conducted using the SPSS 16 software, and *p* ≤ 0.05 will be considered statistically significant.

### Ethical aspects of the study

The present study was approved by the Ethics Committee of Shiraz University of Medical Sciences, Shiraz, Iran (IR.SUMS.REC.1398.664). Informed consent will be obtained from all participants, and confidentiality of the information will be assured.

## Discussion

This study aims to develop a web application and to determine its impact on stress management among college students. Development of the suggested web application and evaluation of its impact on the students will have the potential to develop useful media for managing stress, improving sleep quality, and providing information about the effectiveness of stress management strategies among college students who have been shown to experience high-stress levels. It has been hypothesized that the web application can reduce the stress level and improve the sleep quality among the target college students. It will also promote students’ healthy behaviors. Considering the fact that mental health services are limited in developing countries, many people cannot access fair resources, and only few applications developed in this field are evidence-based. Therefore, this theory-based web application is expected to be a promising tool to help students in Iran. To avoid the risk of contamination between the intervention and control groups, the web application will be available only to the intervention group by putting all intervention group participants in a white list and registering them in the web application. Then, web application registration will be disabled temporarily during the intervention, so that it cannot be used by anyone else. Overall, the study results will indicate an important step towards developing a technology platform for delivering a range of psychosocial strategies for managing stress and sleep problems.

### Trial status

This trial was registered in the Iranian Registry of Clinical Trials (IRCT20160427027647N2) on 14 May 2020. The recruitment began on 14 June 2020 and ended on 05 September 2020.

## Data Availability

Not applicable.

## Abbreviations

ACT: Acceptance and commitment therapy
SRT: Stress Reduction Theory
PSS: Perceived Stress Scale
PSQI: Pittsburgh Sleep Quality Index
